# Supplementary material for: HDL-cholesterol concentration and its association with coronary artery calcification: a systematic review and meta-analysis
Source: Lipids Health Dis. 2023 May 8;22:60. doi: 10.1186/s12944-023-01827-x (PMC10165789; doi:10.1186/s12944-023-01827-x)
Supplement: Supplementary file 1 — Supplementary Material 1 [file 12944_2023_1827_MOESM1_ESM.docx]

**Appendix I**

| **First Author** | **Year** | **Selection** | | | **Comparability** | **Outcome** | | **Score** |
| --- | --- | --- | --- | --- | --- | --- | --- | --- |
| Abd Alamir, M. | 2018 | * | * | * | ** | * |  | 6 |
| Al Rifai, M. | 2018 |  | * | * | ** | * | * | 6 |
| Al Rifai, M. | 2022 | * | * | * | ** | * |  | 6 |
| Allison, M. A. | 2005 | * | * | * | ** | * | * | 7 |
| Bittencourt, M. S. | 2017 | * | * | * | ** | * | * | 7 |
| Chiu, T. Y. | 2012 |  | * | * | ** | * | * | 6 |
| Den Harder, A. M. | 2018 | * | * | * | ** | * |  | 6 |
| Ditah, C. | 2016 | * | * | * | ** | * | * | 7 |
| Freitas, W. M. | 2015 |  | * | * | ** | * | * | 6 |
| Generoso, G. | 2019 | * | * | * | ** | * | * | 7 |
| Hirata, A. | 2020 |  | * | * | ** | * | * | 6 |
| Hisamatsu, T. | 2014 |  | * | * | ** | * | * | 6 |
| Kaplan, H. | 2017 | * | * | * | * | * |  | 5 |
| Kim, J.D. | 2017 | * | * | * | * | * | * | 7 |
| Kimani, C. | 2019 |  | * | * | ** | * | * | 6 |
| Lee, J. | 2020 | * | * | * | ** | * | * | 7 |
| Martin, S. S. | 2011 | * | * | * | ** | * |  | 6 |
| Paramsothy, P. | 2010 | * | * | * | ** | * |  | 6 |
| Pedrosa, J. F. | 2019 | * | * | * | ** | * | * | 7 |
| Pletcher, M. J. | 2013 | * | * | * | ** | * | * | 7 |
| Sharma, A. | 2011 |  | * | * | ** | * | * | 6 |
| Sung, K. C. | 2013 |  | * | * | ** | * | * | 6 |
| Swabe, G. | 2021 |  | * | * | ** | * | * | 6 |
| Wang, J. S. | 2022 |  | * | * | ** | * | * | 6 |
| Woodard, G. A. | 2011 |  | * | * | ** | * | * | 6 |

**NOS score of cross-sectional studies:**

**NOS score of cohort studies:**

| **First Author** | **Year** | **Selection** | | | | **Comparability** | **Outcome** | | | **Score** |
| --- | --- | --- | --- | --- | --- | --- | --- | --- | --- | --- |
| Cardoso, R. | 2020 | * | * | * | * | * | * | * | * | 8 |
| Chandra, A. | 2015 | * | * | * | * | ** |  | * | * | 8 |
| Diederichsen, S. Z. | 2017 | * | * | * | * | ** |  | * | * | 8 |
| El Khoudary, S | 2021 |  | * | * | * | ** | * | * |  | 7 |
| Erbel, R. | 2013 | * | * | * | * | ** | * | * | * | 9 |
| Gao,T. | 2022 | * | * | * | * | ** | * | * | * | 9 |
| Kuller, L. H. | 1999 |  | * | * | * | ** | * | * |  | 7 |
| Mahoney, L. T. | 1996 | * | * | * | * | * | * | * | * | 8 |
| Pletcher, M. J. | 2010 | * | * | * | * | ** | * | * | * | 9 |
| Razavi, A.C. | 2022 | * | * | * | * | ** | * | * | * | 9 |
| Shen, Y. W. | 2020 |  | * | * | * | ** | * | * | * | 9 |
| Wong, N. D. | 2004 |  | * | * | * | ** | * | * | * | 8 |
| Zeb, I. | 2021 | * | * | * | * | ** |  | * | * | 8 |

**Appendix II**

**Search strategy**

**PubMed:**

| ID | Query |  | Results |
| --- | --- | --- | --- |
| #29 | Search ((((((((#7)) AND ((((#12))) AND (#13)) NOT ((((((((((((((#28))) | | 416 |
| #28 | Search (((((((((((((#14) OR (#15)) OR (#16)) OR (#17)) OR (#18)) OR (#19)) OR (#20)) OR (#21)) OR (#22)) OR (#23)) OR (#24)) OR (#25)) OR (#26)) OR (#27) | | 1,063,503 |
| #27 | Search Rheumatoid Arthritis[TIAB] | | 120,193 |
| #26 | Search "Arthritis, Rheumatoid"[Mesh] | | 124,647 |
| #25 | Search Systemic Lupus Erythematosus[TIAB] | | 58,166 |
| #24 | Search “Lupus Erythematosus, Systemic” [Mesh] | | 66,463 |
| #23 | Search Human Immunodeficiency Virus[TIAB] OR Immunodeficiency Virus, Human[TIAB] OR AIDS Virus[TIAB] | | 97,459 |
| #22 | Search “HIV” [Mesh] | | 106,968 |
| #21 | Search Dialyses, Renal[TIAB] OR Dialysis, Renal[TIAB] OR Renal Dialysis[TIAB] OR Hemodialysis[TIAB] OR Hemodialysis[TIAB] | | 119,620 |
| #20 | Search “Renal Dialysis”[Mesh] | | 125,227 |
| #19 | Search End-stage Kidney Disease[TIAB] OR Disease, End-stage Kidney[TIAB] OR End stage Kidney Disease[TIAB] OR Kidney Disease, End-stage[TIAB] OR End stage Renal Disease[TIAB] OR ESRD[TIAB] | | 48,338 |
| #18 | Search “Kidney Failure, Chronic”[Mesh] | | 100,458 |
| #17 | Search Disease, Kidney[TIAB] OR Diseases, Kidney[TIAB] OR Kidney Disease[TIAB] | | 102,679 |
| #16 | Search “Kidney Diseases”[Mesh] | | 568,640 |
| #15 | Search “Chronic Kidney Disease”[TIAB] OR “CKD”[TIAB] | | 75,052 |
| #14 | Search “Renal Insufficiency, Chronic” [Mesh] | | 132,812 |
| #13 | Search Coronary Artery calcification[TIAB] OR Coronary Artery calcification score[TIAB] OR Coronary Artery calcium score[TIAB] OR Coronary Artery calcium[TIAB] OR Coronary Artery calcium scoring[TIAB] OR Coronary calcium score[TIAB] OR CAC Score[TIAB] OR Calcific Coronary Artery Disease[TIAB] OR Calcified Coronary Artery Disease[TIAB] OR coronary arterial calcification[TIAB] OR calcific coronary disease[TIAB] OR calcified coronary artery[TIAB] OR calcifying coronary artery[TIAB] | | 8,792 |
| #12 | Search (((#8) OR (#9) OR (#10) OR (#11) | | 113,242 |
| #11 | Search High-density lipoprotein cholesterol[TIAB] OR HDL-C[TIAB] OR High Density Lipoprotein Cholesterol[TIAB] OR Cholesterol, HDL[TIAB] | | 44,163 |
| #10 | Search “Cholesterol, HDL” [Mesh] | | 31,102 |
| #9 | Search High-Density Lipoproteins[TIAB] OR High Density Lipoproteins[TIAB] OR High Density Lipoprotein[TIAB] OR HDL[TIAB] OR HDL Lipoproteins[TIAB] OR Lipoproteins, HDL[TIAB] OR Lipoprotein, High-Density[TIAB] OR Lipoproteins, High-Density[TIAB] OR Heavy Lipoproteins[TIAB] OR Lipoproteins, Heavy[TIAB] OR Density Lipoprotein, High[TIAB] OR Lipoprotein, High Density[TIAB] | | 102,676 |
| #8 | Search “Lipoproteins, HDL” [Mesh] | | 47,417 |
| #7 | Search (((((#1) OR (#2)) OR (#3)) OR (#4)) OR (#5)) OR (#6) | | 2,773,150 |
| #6 | Search Disease, Coronary[TIAB] OR Diseases, Coronary*[TIAB] OR Coronary Diseases[TIAB] OR Coronary Heart Disease[TIAB] OR Coronary Heart Diseases[TIAB] OR Coronary Heart Diseases[TIAB] OR Disease, Coronary Heart[TIAB] OR Diseases, Coronary Heart[TIAB] OR Heart Disease, Coronary[TIAB] OR Heart Diseases, Coronary[TIAB] | | 57,828 |
| #5 | Search ‘’Coronary Disease’’[Mesh] | | 234,597 |
| #4 | Search Cardiovascular Disease[TIAB] OR Disease, Cardiovascular[TIAB] OR Diseases, Cardiovascular[TIAB] | | 167,924 |
| #3 | Search "Cardiovascular Diseases"[Mesh] | | 2,692,278 |
| #2 | Search Artery Disease, Coronary[TIAB] OR Artery Diseases, Coronary*[TIAB] OR Coronary Artery Diseases[TIAB] OR Disease, Coronary Artery*[TIAB] OR Diseases, Coronary Artery[TIAB] OR Coronary Arteriosclerosis[TIAB] OR Arterioscleroses, Coronary[TIAB] OR Coronary Arterioscleroses[TIAB] OR Atherosclerosis, Coronary[TIAB] OR Atheroscleroses, Coronary[TIAB] OR Coronary Atheroscleroses[TIAB] OR Coronary Atherosclerosis[TIAB] OR Arteriosclerosis, Coronary[TIAB] | | 96,308 |
| #1 | Search "Coronary Artery Disease"[Mesh] | | 74,564 |

**Embase:**

| ID | Query | Results |
| --- | --- | --- |
| #1 | (“Coronary Artery Disease”:ab,ti OR “Artery Disease, Coronary”:ab,ti OR “Artery Diseases, Coronary*”:ab,ti OR “Coronary Artery Diseases”:ab,ti OR “Disease, Coronary Artery*”:ab,ti OR “Diseases, Coronary Artery”:ab,ti OR “Coronary Atherosclerosis”:ab,ti OR “Arterioscleroses, Coronary”:ab,ti OR “Coronary Arterioscleroses”:ab,ti OR “Atherosclerosis, Coronary”:ab,ti OR “Atheroscleroses, Coronary”:ab,ti OR “Coronary Atheroscleroses”:ab,ti OR “Coronary Arteriosclerosis”:ab,ti OR “Arteriosclerosis, Coronary”:ab,ti OR “Cardiovascular Diseases”:ab,ti OR “Cardiovascular Disease”:ab,ti OR “Disease, Cardiovascular”:ab,ti OR “Diseases, Cardiovascular”:ab,ti OR “Coronary Disease”:ab,ti OR “Disease, Coronary”:ab,ti OR “Diseases, Coronary*”:ab,ti OR “Coronary Diseases”:ab,ti OR “Coronary Heart Disease”:ab,ti OR “Coronary Heart Diseases”:ab,ti OR “Disease, Coronary Heart”:ab,ti OR “Diseases, Coronary Heart”:ab,ti OR “Heart Disease, Coronary”:ab,ti OR “Heart Diseases, Coronary”:ab,ti) | 527,314 |
| #2 | (“Lipoproteins, HDL”:ab,ti OR “High-Density Lipoproteins”:ab,ti OR “High Density Lipoproteins”:ab,ti OR “High Density Lipoprotein”:ab,ti OR “HDL”:ab,ti OR “HDL Lipoproteins”:ab,ti OR “Lipoproteins, HDL”:ab,ti OR “Lipoprotein, High-Density”:ab,ti OR “Lipoproteins, High-Density”:ab,ti OR “Heavy Lipoproteins”:ab,ti OR “Lipoproteins, Heavy”:ab,ti OR “Density Lipoprotein, High”:ab,ti OR “Lipoprotein, High Density”:ab,ti OR “Cholesterol, HDL”:ab,ti OR “High-density lipoprotein cholesterol”:ab,ti OR “HDL-C”:ab,ti OR “High Density Lipoprotein Cholesterol”:ab,ti OR “Cholesterol, HDL”:ab,ti) | 147,790 |
| #3 | (“Coronary Artery calcification”:ab,ti OR “Coronary Artery calcification score”:ab,ti OR “Coronary Artery calcium score”:ab,ti OR “Coronary Artery calcium”:ab,ti OR “Coronary Artery calcium scoring”:ab,ti OR “CAC Score”:ab,ti OR “Coronary calcium score”:ab,ti OR “Calcific Coronary Artery Disease”:ab,ti OR “Calcified Coronary Artery Disease”:ab,ti OR “Calcified Coronary Artery Disease”:ab,ti OR “coronary arterial calcification”:ab,ti OR “calcific coronary disease”:ab,ti OR “calcified coronary artery”:ab,ti OR “calcifying coronary artery”:ab,ti) | 11,566 |
| #4 | (“Chronic Kidney Disease”:ab,ti OR “Chronic Kidney Diseases”:ab,ti OR “CKD”:ab,ti OR “Kidney Diseases”:ab,ti OR “Kidney Disease”:ab,ti OR “Disease, Kidney”:ab,ti OR “Diseases, Kidney”:ab,ti OR “Kidney Failure, Chronic”:ab,ti OR “End-stage Kidney Disease”:ab,ti OR “ESRD”:ab,ti OR “Disease, End-stage Kidney”:ab,ti OR “End stage Kidney Disease”:ab,ti OR “Kidney Disease, End-stage”:ab,ti OR “End stage Renal Disease”:ab,ti OR “Renal Dialysis”:ab,ti OR “Dialyses, Renal”:ab,ti OR “Dialysis, Renal”:ab,ti OR “Renal Dialysis”:ab,ti OR “Hemodialysis”:ab,ti OR “Hemodialysis”:ab,ti OR “HIV”:ab,ti OR “Human Immunodeficiency Virus”:ab,ti OR “Immunodeficiency Virus, Human”:ab,ti OR “AIDS Virus”:ab,ti OR “Systemic Lupus Erythematosus”:ab,ti OR “Lupus Erythematosus, Systemic”:ab,ti OR “Rheumatoid Arthritis”:ab,ti OR “Arthritis, Rheumatoid”:ab,ti) | 1,020,960 |
| #5 | #1 AND #2 AND #3 NOT #4 | 543 |

**WOS:**

| ID | Query | Results |
| --- | --- | --- |
| #1 | (TS=("Coronary Artery Disease") OR TS=("Artery Disease, Coronary") OR TS=("Artery Diseases, Coronary") OR TS=("Coronary Artery Diseases") OR TS=("Disease, Coronary Artery") OR TS=("Diseases, Coronary Artery") OR TS=("Coronary Arteriosclerosis") OR TS=("Arterioscleroses, Coronary") OR TS=("Coronary Arterioscleroses") OR TS=("Atherosclerosis, Coronary") OR TS=("Atheroscleroses, Coronary") OR TS=("Coronary Atheroscleroses") OR TS=("Coronary Atherosclerosis") OR TS=("Arteriosclerosis, Coronary") OR TS=("Cardiovascular Diseases") OR TS=("Cardiovascular Disease") OR TS=("Disease, Cardiovascular") OR TS=("Diseases, Cardiovascular") OR TS=("Coronary Disease") OR TS=("Disease, Coronary") OR TS=("Diseases, Coronary") OR TS=("Coronary Diseases") OR TS=("Coronary Heart Disease") OR TS=("Coronary Heart Diseases") OR TS=("Disease, Coronary Heart") OR TS=("Diseases, Coronary Heart") OR TS=("Heart Disease, Coronary") OR TS=("Heart Diseases, Coronary")) | 525,859 |
| #2 | (TS=("Lipoproteins, HDL") OR TS=("High-Density Lipoproteins") OR TS=("High Density Lipoproteins") OR TS=("High Density Lipoprotein") OR TS=("HDL") OR TS=("HDL Lipoproteins") OR TS=("Lipoproteins, HDL") OR TS=("Lipoprotein, High-Density") OR TS=("Lipoproteins, High-Density ") OR TS=("Heavy Lipoproteins") OR TS=("Lipoproteins, Heavy") OR TS=("Density Lipoprotein, High") OR TS=("Lipoprotein, High Density") OR TS=("Cholesterol, HDL") OR TS=("High-density lipoprotein cholesterol") OR TS=("HDL-C") OR TS=("High Density Lipoprotein Cholesterol") OR TS=("Cholesterol, HDL")) | 113,343 |
| #3 | (TS=("Coronary Artery Calcification") OR TS=("Coronary Artery Calcification Score") OR TS=("Coronary Artery Calcium Score") OR TS=("Coronary Artery Calcium") OR TS=("Coronary Artery Calcium Scoring") OR TS=("CAC Score") OR TS=("Coronary Calcium Score") OR TS=("Calcific Coronary Artery Disease") OR TS=("Calcified Coronary Artery Disease") OR TS=("Calcified Coronary Artery Disease") OR TS=("Coronary Arterial Calcification") OR TS=("Calcific Coronary Disease") OR TS=("Calcified Coronary Artery") OR TS=("Calcifying Coronary Artery")) | 10,970 |
| #4 | (TS=("Chronic Kidney Disease") OR TS=("CKD") OR TS=("Kidney Diseases") OR TS=("Disease, Kidney") OR TS=("Diseases, Kidney") OR TS=("Kidney Disease") OR TS=("Kidney Failure, Chronic") OR TS=("End-stage Kidney Disease") OR TS=("Disease, End-stage Kidney") OR TS=("End stage Kidney Disease") OR TS=("Kidney Disease, End-stage") OR TS=("End stage Renal Disease") OR TS=("ESRD") OR TS=("Renal Dialysis") OR TS=("Dialyses, Renal") OR TS=("Dialysis, Renal") OR TS=("Renal Dialysis") OR TS=("Hemodialysis") OR TS=("HIV") OR TS=("Human Immunodeficiency Virus") OR TS=("Immunodeficiency Virus, Human") OR TS=("AIDS Virus") OR TS=("Systemic Lupus Erythematosus") OR TS=("Lupus Erythematosus, Systemic") OR TS=("Rheumatoid Arthritis") OR TS=("Arthritis, Rheumatoid")) | 974,480 |
| #5 | #1 AND #2 AND #3 NOT #4 | 334 |

**Scopus:**

| ID | Query | Results |
| --- | --- | --- |
| #1 | (TITLE-ABS-KEY("Coronary Artery Disease") OR TITLE-ABS-KEY("Artery Disease, Coronary") OR TITLE-ABS-KEY("Artery Diseases, Coronary") OR TITLE-ABS-KEY("Coronary Artery Diseases") OR TITLE-ABS-KEY("Disease, Coronary Artery") OR TITLE-ABS-KEY("Diseases, Coronary Artery") OR TITLE-ABS-KEY("Coronary Arteriosclerosis") OR TITLE-ABS-KEY("Arterioscleroses, Coronary") OR TITLE-ABS-KEY("Coronary Arterioscleroses") OR TITLE-ABS-KEY("Atherosclerosis, Coronary") OR TITLE-ABS-KEY("Atheroscleroses, Coronary") OR TITLE-ABS-KEY("Coronary Atheroscleroses") OR TITLE-ABS-KEY("Coronary Atherosclerosis") OR TITLE-ABS-KEY("Arteriosclerosis, Coronary") OR TITLE-ABS-KEY("Cardiovascular Diseases") OR TITLE-ABS-KEY("Cardiovascular Disease") OR TITLE-ABS-KEY("Disease, Cardiovascular") OR TITLE-ABS-KEY("Diseases, Cardiovascular") OR TITLE-ABS-KEY("Coronary Disease") OR TITLE-ABS-KEY("Disease, Coronary") OR TITLE-ABS-KEY("Diseases, Coronary*") OR TITLE-ABS-KEY("Coronary Diseases") OR TITLE-ABS-KEY("Coronary Heart Disease") OR TITLE-ABS-KEY("Coronary Heart Diseases") OR TITLE-ABS-KEY("Disease, Coronary Heart") OR TITLE-ABS-KEY("Diseases, Coronary Heart") OR TITLE-ABS-KEY("Heart Disease, Coronary") OR TITLE-ABS-KEY("Heart Diseases, Coronary")) | 778,020 |
| #2 | (TITLE-ABS-KEY("Lipoproteins, HDL") OR TITLE-ABS-KEY("High-Density Lipoproteins") OR TITLE-ABS-KEY("High Density Lipoproteins") OR TITLE-ABS-KEY("High Density Lipoprotein") OR TITLE-ABS-KEY("HDL") OR TITLE-ABS-KEY("HDL Lipoproteins") OR TITLE-ABS-KEY("Lipoproteins, HDL") OR TITLE-ABS-KEY("Lipoprotein, High-Density") OR TITLE-ABS-KEY("Lipoproteins, High-Density") OR TITLE-ABS-KEY("Heavy Lipoproteins") OR TITLE-ABS-KEY("Lipoproteins, Heavy") OR TITLE-ABS-KEY("Density Lipoprotein, High") OR TITLE-ABS-KEY("Lipoprotein, High Density") OR TITLE-ABS-KEY("Cholesterol, HDL") OR TITLE-ABS-KEY("High-density lipoprotein cholesterol") OR TITLE-ABS-KEY("HDL-C") OR TITLE-ABS-KEY("High Density Lipoprotein Cholesterol") OR TITLE-ABS-KEY("Cholesterol, HDL")) | 202,519 |
| #3 | (TITLE-ABS-KEY("Coronary Artery calcification") OR TITLE-ABS-KEY("Coronary Artery calcification score") OR TITLE-ABS-KEY("Coronary Artery calcium score") OR TITLE-ABS-KEY("Coronary Artery calcium") OR TITLE-ABS-KEY("Coronary Artery calcium scoring") OR TITLE-ABS-KEY("CAC Score") OR TITLE-ABS-KEY("Coronary calcium score") OR TITLE-ABS-KEY("Calcific Coronary Artery Disease") OR TITLE-ABS-KEY("Calcified Coronary Artery Disease") OR TITLE-ABS-KEY("coronary arterial calcification") OR TITLE-ABS-KEY("calcific coronary disease") OR TITLE-ABS-KEY("calcified coronary artery") OR TITLE-ABS-KEY("Calcifying coronary artery")) | 12,397 |
| #4 | (TITLE-ABS-KEY("Chronic Kidney Disease") OR TITLE-ABS-KEY("CKD") OR TITLE-ABS-KEY("Kidney Diseases") OR TITLE-ABS-KEY("Kidney Disease") OR TITLE-ABS-KEY("Disease, Kidney") OR TITLE-ABS-KEY("Diseases, Kidney") OR TITLE-ABS-KEY("Kidney Failure, Chronic") OR TITLE-ABS-KEY("End-stage Kidney Disease") OR TITLE-ABS-KEY("Disease, End-stage Kidney") OR TITLE-ABS-KEY("End stage Kidney Disease") OR TITLE-ABS-KEY("Kidney Disease, End-stage") OR TITLE-ABS-KEY("End stage Renal Disease") OR TITLE-ABS-KEY("ESRD") OR TITLE-ABS-KEY("Renal Dialysis") OR TITLE-ABS-KEY("Dialyses, Renal") OR TITLE-ABS-KEY("Dialysis, Renal") OR TITLE-ABS-KEY("Renal Dialysis") OR TITLE-ABS-KEY("Hemodialysis") OR TITLE-ABS-KEY("HIV") OR TITLE-ABS-KEY("Human Immunodeficiency Virus") OR TITLE-ABS-KEY("Immunodeficiency Virus, Human") OR TITLE-ABS-KEY("AIDS Virus") OR TITLE-ABS-KEY("Systemic Lupus Erythematosus") OR TITLE-ABS-KEY("Lupus Erythematosus, Systemic") OR TITLE-ABS-KEY("Rheumatoid Arthritis") OR TITLE-ABS-KEY("Arthritis, Rheumatoid")) | 1,272,864 |
| #5 | #1 AND #2 AND #3 NOT #4 | 1,110 |

**Appendix III**

**Sensitivity analysis separated by subgroups (cross-sectional studies):**

mg/dl, CAC>0 ****

Per 1 SD increase and CAC>0

**Sensitivity analysis (Cohort):**

**Appendix IV**

**Publication Bias:**

**Appendix V**

**Abbreviations used in main text, tables, and figures:**

| ALT | Alanine transaminase |
| --- | --- |
| AST | Aspartate aminotransferase |
| BEHHS | Baptist Employee Healthy Heart Study |
| BMI | Body Mass Index |
| BUN | Blood Urea Nitrogen |
| CAC | Coronary Artery Calcification |
| CARDIA | Coronary Artery Risk Development in Young Adults |
| CRP | C-reactive Protein |
| CT | Computed Tomography |
| CVD | Cardiovascular disease |
| ELSA-BRASIL | Brazilian Longitudinal Study of Adult Health |
| FBS | Fasting Blood Sugar |
| GFR | Glomerular Filtration Rate |
| GGT | Gamma-glutamyl transferase |
| HbA1c | Hemoglobin A1C |
| HDL2-C | HDL-C subclass 2 |
| HDL3-C | HDL-C subclass 3 |
| HDL-C | High-density lipoprotein-cholesterol |
| HDL-P | HDL-particle |
| HR | Hazard Ratio |
| IHD | Ischemic Heart Disease |
| IRR | Incident Rate Ratio |
| LDH | Lactate dehydrogenase |
| LDL-C | Low-density lipoprotein-cholesterol |
| LDL-P | LDL-particle |
| LOX-1 | Lectin-like oxidized LDL receptor-1 |
| MASALA | Mediators of Atherosclerosis in South Asians Living in America |
| MCV | Mean Corpuscular Volume |
| MESA | Multiethnic Study of Atherosclerosis |
| MESH | Medical Subject Headings |
| MOOSE | Meta-analysis of Observational Studies in Epidemiology |
| NOS | Newcastle-Ottawa Scale |
| OR | Odds Ratio |
| PR | Prevalence Ratio |
| PRISMA | Preferred Reporting Items for Systematic Reviews and Meta-Analyses |
| PROSPERO | Prospective register of systematic reviews |
| RR | Relative Risk |
| SD | Standard Deviation |
| SESSA | Shiga Epidemiological Study of Subclinical Atherosclerosis |
| SIRCA | Study of Inherited Risk of Coronary Atherosclerosis |
| SWAN | Study of Women’s Health Across the Nation |
| TG | Triglyceride |
| UPOD | Utrecht Patient Oriented Database |
| VLDL-P | Very low-density lipoprotein-particle |
| WBC | White blood cell |
