# Supplementary material for: HDL-cholesterol concentration and its association with coronary artery calcification: a systematic review and meta-analysis
Source: Lipids Health Dis. 2023 May 8;22:60. doi: 10.1186/s12944-023-01827-x (PMC10165789; doi:10.1186/s12944-023-01827-x)
Supplement: Supplementary file 2 — Supplementary Material 2 [file 12944_2023_1827_MOESM2_ESM.pdf]

ithenticate_81052042.docx

WORD COUNT 5203
TIME SUBMITTED 18-APR-2023 02:57PM
PAPER ID 98865489

HDL-cholesterol concentration and its association with coronary artery calcification: a systematic review and meta-analysis

Running title: HDL-cholesterol and coronary artery calcification

Abstract

Background: Coronary artery calcification (CAC) is a potential risk marker of coronary atherosclerosis that has high specificity and sensitivity. However, the association between high-density lipoprotein cholesterol (HDL-C) concentration and CAC incidence and progression is controversial.

Methods: PubMed, Embase, Web of Science, and Scopus were systematically searched to identify relevant observational studies up to March 2023 and assessed the methodological quality using Newcastle-Ottawa Scale (NOS) scale. Random-effects meta-analysis was used to estimate pooled odds ratios (OR) and 95% confidence interval considering heterogeneity across studies.

Results: Of the 2,411 records, 25 cross-sectional (n=71,190) and 13 cohort (n=25,442) studies were included in the systematic review. Ten cross-sectional and eight cohort studies were not eligible and were omitted from the meta-analysis. A total of 15 eligible cross-sectional studies (n=33,913) were included in the meta-analysis and pooled results revealed no significant association between HDL-C and CAC >0, CAC >10, or CAC >100 [pooled OR: 0.99 (0.97, 1.01)]. Meta-analysis of the 5 eligible prospective cohort studies (n=10,721) revealed no significant protective effect of high HDL-C against CAC >0 [pooled OR: 1.02 (0.93, 1.13)].

Conclusions: According to this analysis of observational studies, high HDL-C levels were not found to predict protection against CAC. These results suggest HDL quality rather than HDL quantity is important for certain aspects of atherogenesis and CAC.

Registration number: CRD42021292077.

Key Words: High-density lipoprotein-cholesterol; HDL-C; coronary artery calcification; CAC, calcium score; HDL function, meta-analysis

1. Introduction

Atherosclerotic cardiovascular disease (CVD) is among the greatest cause of morbidity and mortality worldwide. Furthermore, ischemic heart disease (IHD) remains a major cause of CVD. Therefore, identifying the presence of coronary atherosclerosis prior to IHD incidence improves risk stratification and modification [1, 2]. Coronary artery calcification (CAC), a risk marker for atherosclerosis, indicates the presence of IHD, regardless of symptoms or risk factors [3]. CAC has been shown to be correlated with calcium deposition in the arterial wall, a very early stage of atherosclerosis and an established surrogate of the atherosclerotic total burden. Thus, CAC has been used as one of the validated and feasible markers of the presence and extent of subclinical coronary atherosclerosis. The presence of CAC can be detected by non-contrast computed tomography (CT) [4, 5]. High CAC scores (CAC >0) were demonstrated to be linked with an increased risk of cardiovascular events and mortality. The Framingham Risk Score and other conventional risk stratification techniques have been shown to be inferior to the CAC score in terms of predicting future cardiac events and all-cause mortality [6]. As a result, American and European prevention guidelines recently assigned a class IIa recommendation for the use of CAC to further risk stratify and select individuals at borderline and intermediate risks of CVD events [7]. However, considering costs and radiation with CT, CAC evaluation is not a routine test in many health-centers. So, determining specific CAC risk factors could help develop better approaches to understand who needs to perform determination of CAC.

Several studies have shown that serum level of high-density lipoprotein cholesterol (HDL-C) is a biomarker of CAC. HDL delays the formation of atherosclerotic lesions and calcification by removing the cholesterol from macrophages within the coronary arterial wall and transports it to the hepatic cells [8]. It is postulated that lower HDL-C is associated with the presence and progression of CAC, whereas high HDL-C has a protective effect against CAC [9, 10]. However, several other research studies, failed to find this relationship [11, 12]. In the current systematic review and meta-analysis, we sought to investigate the relationship between HDL-C and the progression and incidence of CAC.

2. Methods

This meta-analysis was conducted in compliance with the recommendations of the Meta-analysis of Observational Studies in Epidemiology (MOOSE) guidelines [13]. The protocol of the present systematic review in the International

Prospective Register of Systematic Reviews (PROSPERO) was documented (registration number: CRD42021292077).

2.1. Search strategy

Online databases, including PubMed, Embase, Web of Science (ISI), and Scopus were searched systematically until March 2023 without language restriction. To enhance the sensitivity and specificity, a combination of Medical Subject Headings (MESH) and non-MESH words was utilized to capture studies. The following keywords were used: (“Coronary Artery Disease”[Mesh] OR “Coronary Arteriosclerosis” OR “Cardiovascular Diseases”[Mesh] OR “Coronary Heart Disease”) AND (“Lipoproteins, HDL” [Mesh] OR “High Density Lipoprotein” OR HDL OR “Heavy Lipoproteins” OR “High-density lipoprotein cholesterol” OR HDL-C OR “High Density Lipoprotein Cholesterol”) AND (“Coronary Artery calcification” OR “Coronary Artery calcification score” OR “Coronary Artery calcium score” OR “Calcific Coronary Artery Disease” OR “calcific coronary disease”). Supplementary File Appendix 2 contains the entire search strategy. We also manually searched the reference lists of all retrieved articles and Google Scholar to identify any overlooked relevant publications.

2.2. Study selection and Eligibility criteria

Three authors (FA, SS, NO) independently screened manuscripts to identify eligible studies and all manuscripts were included based on consensus among all authors. According to the PECOT (Population, Exposure, Comparison, Outcome, Type of study) template, the study inclusion criteria included (P) human samples, (E and C) examining the association of low or high levels of HDL-C, (O) on CAC score, (T) in observational studies (cohort, case-control, or cross-sectional). The following studies were excluded: studies with absence of HDL-C or its adjusted association with CAC, duplicate samples (studies based on the same sample/population), review articles, editorials, clinical guidelines, personal opinions, book chapters, conference abstracts, case reports, genetic studies, animal studies, and studies focusing on diseases other than coronary artery disease.

2.3. Data extraction and Quality assessment

Data were independently extracted from included studies by three authors (FA, SS, NO) based on consensus among all authors. The following data was extracted: first authors, publication years, data source, study types, participants information (gender, age of patients, geographical location, sample size, and basic diseases), outcome definition,

outcome measures including effect sizes and risk estimates (Odds Ratios) with their confidence intervals, stratification based on controlled variable in the multivariable model, duration of follow-up (for cohort studies), and quality assessment.

The methodological quality of evidence of each study was assessed using the Newcastle-Ottawa Scale (NOS) as previously established based on stringent criteria related to the 4 domains of selection, comparability, exposure (cross-sectional studies) or outcome (cohort studies) [14]. In general, an article score ≥ 7, is considered to be of good quality [14].

2.4. Statistical analysis

The observed relationship between HDL-C and CAC was estimated using Odds Ratios (ORs) as the effect size. Random effects meta-analysis was conducted to obtain the pooled OR and its 95% confidence intervals using the Der-Simonian and Laird method. A random-effects meta-analysis was used to account for conceptual and clinical heterogeneity between studies with a forest plot to demonstrate the ORs and respective 95% confidence intervals.

To assess study heterogeneity, the I^2 statistic ($I^2 \geq 50\%$ indicates substantial heterogeneity) was utilized. Also, Cochran's Q statistic was utilized with a significance level of $P < 0.10$ to indicate the variance among studies.

Sensitivity analysis with serial removal of a specific study or group of studies assessed the robustness of the pooled results. A subgroup meta-analysis on the association between HDL-C and CAC was conducted by HDL-C measurement scale (mg/dl vs. per 1 standard deviation increase).

To assess the publication bias, visual inspection of funnel plots was performed, so as log ORs were plotted against their standard errors (as study precision). Also, the Egger's regression asymmetry test and Begg's adjusted rank correlation test were performed. Statistical tests were two-tailed and significance levels were considered less than 0.10 for analyses. All statistical analyses were performed using the Stata version 14 software (Stata Corp., College Station, TX, USA).

3. Results

3.1. Search results

The flowchart of literature search and selection process is presented in Fig. 1. Using systematic database searching, 2,403 potentially relevant publications were identified in the first evaluation, as well as eight studies, through a manual search of the reference lists of these papers. Subsequently, 1,141 duplicates and 1,109 irrelevant articles after screening titles and abstracts. The full texts of 161 potentially relevant publications were evaluated to determine if they met the eligibility criteria. Of these, 123 publications were excluded for the reasons mentioned in the Preferred Reporting Items for Systematic Reviews and Meta-Analyses (PRISMA) flow diagram. Finally, 38 articles met the eligibility criteria and were involved in the qualitative synthesis (systematic review) [9-12, 15-48] and 20 were included in the quantitative synthesis (meta-analysis) [9, 11, 12, 16, 20, 22-24, 26-28, 32, 34, 35, 39-41, 45, 47, 48].

3.2. Association between HDL-C and CAC in cross-sectional studies

3.2.1. Study Characteristics

Twenty-five included cross-sectional studies [9, 11, 12, 15-35, 47] enrolled 71,190 participants and the sample size ranged from 104 to 16,493 participants. Populations varied in sex distribution and age, and also had mean or median HDL-C levels. Nineteen studies included both genders [9, 11, 12, 15-22, 25, 26, 28-32, 47], four included only men [23, 24, 27, 33], and two included only women [34, 35]. The average age of study participants varied (22-94 years). Of the 25 included cross-sectional studies, 14 were conducted in America [9, 11, 15-18, 21, 22, 25, 29-31, 34, 35], one in Europe [19], and ten in Asia [12, 20, 23, 24, 26-28, 32, 33, 47]. The sources of the three studies were the Multiethnic Study of Atherosclerosis (MESA) [15, 30, 31], three were from the Brazilian Longitudinal Study of Adult Health (ELSA-BRASIL) [9, 11, 22], three were from Shiga Epidemiological Study of Subclinical Atherosclerosis (SESSA) [23, 24, 27], and two studies were from the Study of Women's Health Across the Nation (SWAN) [34, 35]. One study was a cross-sectional study of both MESA and ELSA-BRASIL [16], and four other studies were based on the Brazilian Study on Healthy Aging [21], Utrecht Patient Oriented Database (UPOD) [19], Study of Inherited Risk of Coronary Atherosclerosis (SIRCA) [29], and Mediators of Atherosclerosis in South Asians Living in America (MASALA) cohorts. All studies were published in English in the last 17 years (2005-2022). Nineteen studies reported an OR estimate for CAC, adjusted at least for age and sex [9, 11, 12, 16, 18, 20-24, 26-28, 31-35, 47]. In six other studies, the association between HDL-C and CAC was reported as relative risk (RR) [30], prevalence ratio (PR) [15], incident rate ratio (IRR) [25], coefficient β [17, 19], or tobit regression [29]. Many studies also adjusted for cardiovascular risk factors and other confounding factors. The presence of CAC was defined as a score greater than

0, 1, 10, or 100. Seventeen studies analyzed HDL-C as a quantitative variable of mg/dL [9, 12, 15, 17, 18, 20, 21, 23, 24, 28, 30-35, 47], two studies as mmol/L [19, 25] and six studies interpreted HDL-C in terms of one standard deviation increase [11, 16, 22, 26, 27, 29]. All included studies were assessed as having moderate to good quality according to the NOS scale (Supplementary Appendix 1). Details of each study are presented in Table 1.

3.2.2. Systematic review findings

Eleven cross-sectional studies reported an inverse relationship between HDL-C levels and CAC incidence [9, 15, 18, 21, 24-26, 29, 31-33]. In other words, higher HDL-C serum levels had a protective role against the presence of CAC. In a cross-sectional of MESA cohort, low HDL-C was associated with higher rates of multivessel CAC (PR: 1.20 (1.02, 1.40), < 0.01) [15]. In another cross-sectional study of the MESA cohort, HDL-C was associated with CAC presence (OR: 0.892 (0.854, 0.931), <0.001) but not with CAC extent (OR: 0.006 (-0.038, 0.05), 0.791) [31]. In a cross-sectional study of the ELSA-Brasil cohort, HDL-C was associated with CAC ≥1 (OR: 0.99 (0.98, 0.99), ≤0.05) [9]. HDL-C level was associated with CAC >0 (OR: 0.56 (0.34, 0.91)) in a cross-sectional study of the SESSA cohort [24]. In a cross-sectional study of the SIRCA cohort, HDL-C was inversely associated with CAC after adjusting for age and sex (tobit ratio: 0.72 (0.59, 0.88), 0.001), but after further adjustment for medications, blood pressure, lipids, tobacco and alcohol use, exercise, family history of premature IHD, body mass index (BMI), waist circumference, and high-sensitivity CRP got insignificant [29]. In a cross-sectional study of the Brazilian Study on Healthy Aging cohort in individuals aged 80 years or over, the association between HDL-C and CAC was significant (OR: 0.34 (0.15, 0.75), 0.008) [21]. In another cross-sectional study, individuals with low HDL-C had higher CAC scores and the adjusted correlation of HDL-C and CAC was significant in both men and women (OR: 0.92 (0.89, 0.95) and 0.93 (0.90, 0.96), respectively) [18]. HDL-C decreased the risk of CAC (IRR: –0.04 (–0.07, 0.00), 0.0474) [25], and it was also associated with CAC in three other cross-sectional studies; (OR: 0.87 (0.82, 0.93), <0.05) [26], (OR: 0.93 (0.87, 0.99), ≤0.0001) [32], and (OR: 0.78 (0.64, 0.94), 0.01) [33].

However, 11 studies failed to demonstrate the protective role of HDL-C against CAC incidence [11, 12, 16, 19, 20, 22, 23, 27, 28, 30, 47]. HDL-C level was not associated with CAC in a cross-sectional study of the MESA cohort (RR: 1.05 (0.98, 1.12)) [30]. HDL-C was also not independently associated with the presence or extent of CAC in a cross-sectionals of ELSA-Brasil cohort (OR: 1.041 (0.933, 1.161), and 0.940 (0.800, 1.105), respectively) [22]. In another cross-sectional study of ELSA-Brasil, lower HDL-C was not associated with CAC (OR: 1.02 (0.93, 1.13), 0.46) [11].

In a cross-sectional of MESA and ELSA-Brasil, adjusted prevalence OR for the association of HDL-C and CAC > 0 was insignificant (0.80 (0.60, 1.07)) [16]. Two cross-sectional studies of SESSA cohort also failed to demonstrate significant association between HDL-C and CAC; (OR: 1.03 (0.86, 1.22), 0.72) [23] and (OR: 0.98 (0.8, 1.2), <0.955) [27]. In a cross-sectional study of UPOD, HDL-C was not associated with CAC (β coefficient: -0.05 (-0.17, 0.14), 0.559) [19]. The association between HDL-C and CAC was also insignificant in four other studies (OR: 0.98 (0.96, 1)) [12], (OR: 0.59 (0.27, 1.29) 0.49) [20], (OR: 0.99 (0.97, 1), <0.094) [28], and (OR: 1.25 (0.97, 1.61), 0.09) [47].

In a recent cross-sectional of MASALA, HDL-C was directly associated with CAC density (β coefficient: 0.009 (0.001, 0.016)) but the reverse association between HDL-C and CAC volume was insignificant (β coefficient: -0.004 (-0.009, 0.001)) [17]. It is suggested that higher HDL-C probably results in denser coronary plaques that are more stable and therefore less likely to rupture.

Two cross-sectional studies investigated the relationship between HDL-C and CAC in menopausal women. In a cross-sectionals study of SWAN cohort, it is revealed that HDL-C is not protective against high CAC or any left main CAC in premenopausal or early perimenopausal (OR: 0.99 (0.95, 1.03) and 1.01 (0.96, 1.05), respectively). Moreover, in the late-perimenopausal or postmenopausal group, HDL-C was not protective against high CAC (0.99 (0.96, 1.02)), but was protective against left main CAC (1.08 (1.00, 1.16)) [35]. In a recent cross-sectional study of the SWAN cohort there was no association between HDL-C and CAC >10 (OR: 1.04 (0.95, 1.12), 0.4) [34].

3.2.3. Meta-analysis findings

Ten cross-sectional studies were omitted from the meta-analysis because their data were not eligible for quantitative synthesis [15, 17-19, 21, 25, 29-31, 33]. Briefly, they have different statistic methodology for reporting association between HDL-C and CAC [15, 19, 25, 29, 31], different analyzing HDL-C variables [17, 18, 30, 33] and one study had included only elderly individuals aged >80 years [21].

Fifteen cross-sectional studies were eligible for the meta-analysis [9, 11, 12, 16, 20, 22-24, 26-28, 32, 34, 35, 47]. Ten studies analyzed HDL-C as a quantitative variable (mg/dL) [9, 12, 20, 23, 24, 28, 32, 34, 35, 47] and five studies measured and interpreted it in terms of one standard deviation increase [11, 16, 22, 26, 27]. The Pooled results revealed no significant association between HDL-C and CAC >0, CAC >10, or CAC >100 (OR: 0.99 (0.97, 1.01)) as illustrated in Fig. 2.

3.3. Association between HDL-C and CAC in cohort studies

3.3.1. Study Characteristics

Thirteen cohort studies [10, 36-46, 48] enrolled 25,442 participants with sample sizes ranging from 21 to 6,011 participants. The duration of follow-up was between 5-20 years. Populations varied in sex distribution and age, and also had mean or median HDL-C levels. Eleven cohorts included both genders [10, 36-38, 40, 41, 43-46, 48] and two included only women [39, 42]. The average age of the participants ranged from 29 to 84 years old. Of the 12 included cohort studies, ten were conducted in America [10, 36, 37, 39, 41-44, 46, 48], two in Europe [38, 40], and one in Asia [45]. All studies were published in English in the last 26 years (1996-2022). Seven studies reported an OR estimate for CAC adjusted at least for age and sex [36, 39-44]. In six other studies, the association between HDL-C and CAC was reported as hazard ratio (HR) [45, 48], RR [10], IRR [38], standardized β [37], or baseline and change in CAC [46]. Many studies also adjusted for cardiovascular risk factors and other confounding factors. The incidence, progression, and density of CAC were investigated in these studies. Eleven studies analyzed HDL-C as a quantitative variable (mg/dL) [10, 37, 39-46, 48], one study as mmol/L [38], and one study had interpreted HDL-C as one standard deviation increase [36]. All included studies were good quality according to the NOS scale (Supplementary Appendix 1). Details of each study are presented in Table 2.

3.3.2. Systematic review findings

In a Muscatine study cohort, decreased HDL-C measured during young adult life was associated with the presence of CAC in young adults (OR: 5.5 (2, 15.2), < 0.001) [43]. In the National Cholesterol Education Program, there was an association between higher HDL-C (> 60 mg/dl) and less progression of CAC volume (change in volume score: 203 (-52, 2,828) for HDL-C ≥ 60, 159 (-123, 3,872) for 40 < HDL-C ≤ 59, and 151 (0–2, 213) for HDL-C ≤ 40; P= 0.03) [46]. In another study, among subjects with a zero CAC score at baseline, there was an independent association between HDL-C and CAC progression (HR: 0.976 (0.953, 0.999), 0.043) [45]. In ELSA-Brasil cohort, lower HDL-C was associated with CAC incidence (OR: 0.83 (0.72, 0.96), 0.01) but not CAC progression (OR: 0.89 (0.74, 1.06), 0.19) [36]. However, in a recent study of MESA, HDL-C was associated with both a low risk of incident CAC development (RR: 0.92 (0.89, 0.96), <0.001) and lower annual CAC progression (difference in average progression β: -0.92 (-1.74, -0.1), <0.027) [10].

In the Coronary Artery Risk Development in Young Adults (CARDIA) cohort, non-optimal HDL-C during young adulthood was associated with CAC two decades later. HDL was independently associated with CAC in this study (OR for average exposure to HDL-C before age 35: 2.8 (1.1, 6.8), 0.03) [44]. In contrast, in a recent CARDIA cohort, there was no association between HDL-C and CAC progression (OR: 0.83 (0.647, 1.065), 0.143) [41]. Another recent cohort of both MESA and CARDIA showed that age might influence the relationship between HDL-C level and CAC. The results revealed a significant association between HDL-C and CAC incidence in middle-aged people between 46 to 64 years old (HR: 1.23 (1.12, 1.34)), but the association was insignificant in young individuals between 32 to 45 (HR: 1.07 (0.96, 1.19)) and older individuals between 65 to 84 years old (HR: 1.10 (0.97, 1.25)) [48]. A few cohorts also reported no association between HDL-C and CAC. In the Dallas Heart Study, HDL-C was not associated with prevalent CAC in adjusted models (*P*= 0.13) [37]. In Heinz Nixdorf Recall Study, HDL-C was not associated with CAC progression in either men or women (OR: 0.93 (0.7, 1.29) and 1.00 (0.78, 1.20), respectively) [40]. In another cohort, there was no association between HDL-C and the incidence (IRR: 0.84 (0.46, 1.54), 0.60) or progression of CAC (IRR: 0.94 (0.60, 1.48), 0.80) [38].

Two cohort studies investigated the relationship between HDL-C and CAC in menopausal women. In Healthy Women Study, HDL-C was associated with CAC progression in postmenopausal women. Adjusted OR for 10 mg/dL increase of HDL-C was 0.53 (0.03, 0.93) in women with CAC $\geq$ 101 [42]. However, in the SWAN cohort, there was no association between HDL-C and the progression of CAC after menopause (OR: 0.78 (0.51, 1.19), 0.60) or CAC density (OR: -1.48 (-10.76, 8.88), 0.77) in women transitioning through menopause [39].

3.3.3. Meta-analysis findings

Eight cohorts were omitted from meta-analysis as their data were not eligible for quantitative synthesis [10, 36-38, 42-44, 46]. They have different statistic methodology for reporting association between HDL-C and CAC [37, 38, 46], different analyzing HDL-C variables [10, 42-44] and one study had included patients with CAC >0 at initiation of cohort [36]. Five cohorts were eligible for meta-analysis [39-41, 45, 48]. The pooled results revealed no significant association between HDL-C and CAC (OR: 1.02 (0.93, 1.13)) as illustrated in Fig. 3. A cumulative forest plot was also created (Fig. 4).

3.4. Sensitivity analysis and publication bias

In cross-sectional studies, sensitivity analysis separated by subgroups was done and revealed that meta-analysis model was robust. Also, sensitivity analysis for cohort studies showed that the association between HDL-C and CAC was consistent (range of summary ORs: 0.99-1.04), indicating that the meta-analysis model was robust (Supplementary Appendix 3). To assess possible publication bias, the association between CAC and HDL-C are presented in a funnel plot (Fig. 5). There was no evidence of publication bias by visual inspection and assessment of statistical tests (*P*= 0.25, for Begg's adjusted rank correlation test and *P*= 0.99 for Egger's regression asymmetry test) (Supplementary Appendix 4).

4. Discussion

In this study, 25 cross-sectional (n=71,190) and 13 cohort (n=25,442) studies were systematically reviewed to characterize the link between CAC and HDL-C. Several studies have reported an independent inverse relationship between serum HDL-C and CAC incidence and progression. However, many other studies failed to reveal a statistically significant relationship between HDL-C and CAC incidence after adjustment for confounding factors. A meta-analysis of 15 eligible cross-sectionals (n=33,913) and five eligible cohort studies (n=10,721) demonstrated an insignificant association between HDL-C and CAC. Subsequently, HDL-C blood level might not be considered an independent risk factor for CAC.

Similarly, although previous epidemiological studies have been extensively demonstrated the inverse relationship between HDL-C plasma levels and IHD with a strong, graded and coherent pattern [49, 50], Mendelian randomization studies then revealed no causal association between HDL-C and the pathogenesis of IHD [51]. In a recent cohort study of 15.8 million Korean adults, both elevated and low HDL-C were associated with elevated mortality from CVD, and high HDL-C serum concentration was not necessarily a sign of better cardiovascular health [52]. Moreover, pharmacological increase in HDL-C with drugs such as fibrates, niacin, or cholesteryl ester transfer protein inhibitors failed to reduce IHD in several clinical trials [53]. Therefore, the HDL-C level is now considered a biomarker of cardiovascular health rather than a risk factor. This controversy might be the result of crude measurement of the total cholesterol content in HDL, while the entity of HDL is characterized by its structure and function [54, 55]. For instance, our previous studies demonstrated that increased HDL lipid peroxidation, which impairs the antioxidant function of HDL, is positively associated with cardiovascular events in the MASHAD cohort [56-59]. It is obvious

that a simple measurement of cholesterol carried by HDL particles does not reflect HDL functionality or composition in the prediction or prevention of IHD. HDL subfractions can be classified according to their size, shape, charge, density, functionality, and biochemical composition. Although HDL-C is the only reproducible and standardized parameter available to estimate plasma concentration of HDL, there was an association between increased levels of small HDL and low proportions of large particles with an increased risk of coronary artery disease [60]. Hence, measurement of specific HDL subfractions would be a better biomarker than HDL-C level to evaluate the risk of IHD [61]. The same is true regarding the association between CAC and HDL-C levels. According to the results of the present meta-analysis, no clinically significant correlations were observed between HDL-C plasma levels and incidence or progression of CAC.

Interestingly, some observational clinical studies have indicated an association between different HDL subfractions and CAC. In cohort of Dallas Heart Study (9.3 years), HDL-particle (HDL-P) were inversely associated with prevalent CAC in fully adjusted models, including risk factors and HDL-C (standardized $\beta = -0.06$, $P = 0.009$). Furthermore, HDL-C was only associated with prevalent CAC after serial adjustment for HDL-P (standardized $\beta = 0.07$, $P = 0.008$) [37]. In a cross-sectional study, HDL-P and medium size HDL-P were protective against CAC (OR: 0.42 (0.22, 0.79), 0.002 and 0.36 (0.19, 0.69), 0.006, respectively), while large HDL-P and average size HDL-P were not (OR: 0.77(0.33, 1.83), 0.29 and 0.72(0.35, 1.48), 0.58, respectively) [20]. While high HDL-P was significantly negatively linked with CAC progress in the MESA cohort (9.6 0.6 years), this association was diminished when conventional lipids were taken into account. Low HDL-P levels were not linked to the development of CAC [62]. Cross-sectional SESSA demonstrated impaired antiatherogenic function of HDL in correlation with the binding capacity of dysfunctional HDL to lectin-like oxidized LDL receptor-1 (LOX-1). The adjusted OR of HDL-P for CAC was not significant in this study (0.92 (0.78, 1.08), 0.33) [23]. In another cross-sectional of SESSA, HDL-P concentrations and size also were not in association with the presence of CAC (OR 1.04 (0.62, 1.75) and 0.66 (0.40, 1.10), respectively) [24]. Similar results were obtained in a cross-sectional study by Mahajan et al. on age-adjusted PR of HDL-P concentrations and size with CAC [63].

HDL size is also a determinant of the anti-atherogenic properties of HDL. In a cross-sectional study of the Baptist Employee Healthy Heart Study (BEHHS) randomized trial, small HDL and large HDL offered modest protection against CAC (OR: 0.92 (0.89, 0.99) and 0.89 (0.83, 0.95), respectively) [64]. In another cross-sectional of Healthy Women Study, large HDL was inversely associated with CAC, but small HDL was not [65]. In a cross-sectional of

MESA, the presence of proinflammatory protein apolipoprotein C-III on HDL was positively associated with CAC, whereas HDL lacking apolipoprotein C-III was inversely associated with CAC [66]. In some studies, HDL-C subclass 2 (HDL2-C), which is composed only of apolipoprotein A-I was more anti-atherogenic than subclass 3 (HDL3-C) which contains both apolipoprotein A-I and apolipoprotein A-II. In cohort of Healthy Women Study, the level of HDL2-C was strongly and inversely related to CAC (r= -0.31, <0.001) and was much stronger in comparison with HDL3-C [42]. Decrease of HDL2-C was significantly associated with the increase of CAC prevalence and extent (OR: 3.45 (2.03, 50.1)), while in a cross-sectional of a preventive cardiology outpatient program cohort, HDL3-C was not (OR: 1.33 (0.07, 26.9)) [67]. But in a cross-sectional of ELSA-Brasil, neither HDL2-C and HDL3-C nor HDL2-C/HDL3-C ratio were independently associated with the presence or extent of CAC after adjustment for epidemiological variables and traditional CVD risk factors [22]. In cohort of SWAN HDL study (before and after menopause), longitudinal associations of the adjusted HDL metrics (total HDL-P, large HDL-P, medium HDL-P, small HDL-P, HDL size, HDL-phospholipid, HDL-triglyceride, HDL-C efflux capacity) with CAC incidence and density were investigated. CAC incidence was only associated with medium HDL-P (OR: 1.46 (1.12, 1.90), 0.006) and small HDL-P (OR: 0.76 (0.58, 1.01), 0.05). CAC density was not associated with any HDL metric in the adjusted model [39]. The HDL content of triglyceride, phospholipid, total cholesterol, and esterified cholesterol of different HDL subclasses were also evaluated in a cross-sectional of Genetics of Atherosclerosis Disease study. The findings showed that HDL subclasses might be CAC markers, however, they do not support an association between lipoproteins and CAC scores [68]. Thus, there is a controversy between the results of HDL particles and subfractions with CAC. This is probably due to the absence of a unique gold standard to measure the functional and physical characteristics of HDL subfractions among studies. Lack of standard and easily applicable methods to analyze and detect HDL particles and subfractions, limits their clinical usefulness for the assessment of cardiovascular risk. Therefore, further studies are needed to fully understand the impact of HDL subfractions and HDL-C in atherosclerotic CVD risk stratification in para clinics and different populations [69].

5. Strengths and limitations

This study had several strengths. First, this is the first systematic review and meta-analysis of HDL-C concentration and CAC in the literature. Second, it has replicable and extensive methods for searching the published literature. Third,

a large sample size study was conducted, including 91,160 individuals from a wide region of Asia, Europe, and America in a cohort or cross-sectional design. Thus, a conclusive result with low bias and high precision was obtained for the general population in the current study. Finally, in addition to resolving the controversy between existing studies on the association between HDL-C level and CAC, this article reveals that HDL-C fails to predict the risk of CAC and should not be used as a biomarker or risk factor for CAC measurement and screening.

The present systematic review and meta-analysis has limitations. First, the included population were a wide range of healthy adults at baseline or a random sample of society with different ages and races. So, the heterogeneity among patients may affect how CAC is found to be linked with HDL-C. Moreover, 35 of the 38 studies were conducted in America and Asia, so the results of this meta-analysis may not be applied to other continents where different lifestyles and races would affect the association. Thus, it is uncertain whether the results are applicable to other ethnicities. Second, HDL-C levels and CAC measurements reported in each included study were measured at different laboratory centers using different assay methods, equipment and experimental kits. In addition, different cutoffs for HDL-C and CAC scores have been proposed. This may have caused inconsistencies in data interpretation. Third, most of the studies included in the meta-analysis were cross-sectional in design and temporal or causal relationships between HDL-C and CAC incidence cannot be determined. Only four cohorts were eligible for the meta-analysis and one study included only midlife women. Therefore, robust subgroup analysis was not feasible. Fourth, although multivariable adjustment was conducted in all included studies, they were not justified for identical confounding factors and may lead to discrepancies. However, we selected studies in meta-analysis that reported an estimate of OR for CAC adjusted at least for age and sex. In addition, all included studies are still subjected to bias, because many unexpected and unknown confounding factors may exist. Finally, as mentioned above, all included studies in meta-analysis measured HDL-C content but did not represent the functionality of HDL in preventing CAC and atherosclerosis.

6. Conclusions

The present meta-analysis findings indicate that high HDL-C levels have no significant protective effects against CAC in cohort and cross-sectional studies. Accordingly, this analysis did not reveal a major role for HDL-C level in CAC. As a result, HDL-C concentration cannot be used as a predictor or risk factor to estimate the need for CAC

measurement and screening. One reason might be that crude measurement of total cholesterol content in HDL, such as HDL-C, does not represent the structure and function of HDL. This finding supports the concept that HDL quality, rather than quantity, is more important for certain aspects of atherogenesis and CAC. Standard measurement of HDL particles and subfractions and its association with CAC should be investigated in future large-scale prospective research to confirm these findings.

ithenticate_81052042.docx

ORIGINALITY REPORT

23%
SIMILARITY INDEX

PRIMARY SOURCES

1	www.ncbi.nlm.nih.gov<br>Internet	97 words — 2%
2	www.science.gov<br>Internet	80 words — 1%
3	Sung, Ki-Chul, Sarah H. Wild, and Christopher D. Byrne. "Controlling for apolipoprotein A-I concentrations changes the inverse direction of the relationship between high HDL-C concentration and a measure of pre-clinical atherosclerosis", Atherosclerosis, 2013.<br>Crossref	77 words — 1%
4	www.researchgate.net<br>Internet	77 words — 1%
5	Anatol Kontush, M. John Chapman. "High‐Density Lipoproteins", Wiley, 2011<br>Crossref	74 words — 1%
6	Amarenco, P.. "High-density lipoprotein-cholesterol and risk of stroke and carotid atherosclerosis: A systematic review", Atherosclerosis, 200802<br>Crossref	68 words — 1%
7	www.mdpi.com<br>Internet	59 words — 1%

8	link.springer.com<br>Internet	54 words — 1%
9	Salim S. Virani, Alvaro Alonso, Hugo J. Aparicio, Emelia J. Benjamin et al. "Heart Disease and Stroke Statistics—2021 Update", Circulation, 2021<br>Crossref	39 words — 1%
10	Chobufo Ditah, James Otvos, Hisham Nassar, Dorith Shaham, Ronit Sinnreich, Jeremy D. Kark. "Small and medium sized HDL particles are protectively associated with coronary calcification in a cross-sectional population-based sample", Atherosclerosis, 2016<br>Crossref	38 words — 1%
11	Alvin Chandra, Ian J. Neeland, Sandeep R. Das, Amit Khera, Aslan T. Turer, Colby R. Ayers, Darren K. McGuire, Anand Rohatgi. "Relation of Black Race Between High Density Lipoprotein Cholesterol Content, High Density Lipoprotein Particles and Coronary Events (from the Dallas Heart Study)", The American Journal of Cardiology, 2015<br>Crossref	34 words — 1%
12	Ling Liang, Xianghua Hou, Kevin R. Bainey, Yanlin Zhang, Wayne Tymchak, Zhongquan Qi, Weihua Li, Hoan Linh Banh. "The association between hyperuricemia and coronary artery calcification development: A systematic review and meta‐analysis", Clinical Cardiology, 2019<br>Crossref	34 words — 1%
13	www.teses.usp.br<br>Internet	33 words — 1%
14	purehost.bath.ac.uk<br>Internet	28 words — < 1%

15	"Full Issue PDF", Journal of the American College of Cardiology, 2018<br>Crossref	27 words — < 1%
16	academic.oup.com<br>Internet	27 words — < 1%
17	observatorio.fm.usp.br<br>Internet	27 words — < 1%
18	dmsjournal.biomedcentral.com<br>Internet	24 words — < 1%
19	"Therapeutic Lipidology", Springer Science and Business Media LLC, 2007<br>Crossref	23 words — < 1%
20	Martin, S.S.. "Comparison of High-Density Lipoprotein Cholesterol to Apolipoprotein A-I and A-II to Predict Coronary Calcium and the Effect of Insulin Resistance", The American Journal of Cardiology, 20110201<br>Crossref	23 words — < 1%
21	Sudipa Sarkar, Sabina Haberlen, Seamus Whelton, Edward Schneider et al. "Greater IL-6, D-dimer, and ICAM-1 Levels Are Associated With Lower Small HDL Particle Concentration in the Multicenter AIDS Cohort Study", Open Forum Infectious Diseases, 2019<br>Crossref	23 words — < 1%
22	Rhanderson Cardoso, Giuliano Generoso, Henrique L. Staniak, Murilo Foppa et al. "Predictors of coronary artery calcium incidence and progression: The Brazilian Longitudinal Study of Adult Health (ELSA-Brasil)", Atherosclerosis, 2020<br>Crossref	22 words — < 1%

23	Ho, P.C.. "Serum lipids in Chinese patients using oral contraceptive pills", Contraception, 199001<br>Crossref	21 words — < 1%
24	Mahmoud Al Rifai, Alka M. Kanaya, Namratha R Kandula, Jaideep Patel et al. "Association of Coronary Artery Calcium Density and Volume with Predicted Atherosclerotic Cardiovascular Disease Risk and Cardiometabolic Risk Factors in South Asians: The Mediators of Atherosclerosis in South Asians Living in America (MASALA) Study", Current Problems in Cardiology, 2022<br>Crossref	21 words — < 1%
25	journals.sagepub.com<br>Internet	20 words — < 1%
26	synapse.koreamed.org<br>Internet	20 words — < 1%
27	discovery.ucl.ac.uk<br>Internet	18 words — < 1%
28	e-jla.org<br>Internet	17 words — < 1%
29	journals.lww.com<br>Internet	17 words — < 1%
30	cdr.lib.unc.edu<br>Internet	16 words — < 1%
31	www.researchsquare.com<br>Internet	16 words — < 1%
32	www.frontiersin.org<br>Internet	15 words — < 1%

33	Giuliano Generoso, Isabela M. Bensenor, Raul D. Santos, Henrique L. Staniak et al. "High-density Lipoprotein-cholesterol Subfractions and Coronary Artery Calcium: The ELSA-Brasil Study", Archives of Medical Research, 2019<br>Crossref	14 words — < 1%
34	jhpn.biomedcentral.com<br>Internet	14 words — < 1%
35	onlinelibrary.wiley.com<br>Internet	14 words — < 1%
36	www.hindawi.com<br>Internet	14 words — < 1%
37	www.jstage.jst.go.jp<br>Internet	14 words — < 1%
38	Alexis Nasr, Karen A. Matthews, Maria M. Brooks, Daniel S. McConnell et al. "Vasomotor symptoms and lipids/lipoprotein subclass metrics in midlife women: Does level of endogenous estradiol matter? The SWAN HDL Ancillary Study", Journal of Clinical Lipidology, 2020<br>Crossref	12 words — < 1%
39	pure.rug.nl<br>Internet	12 words — < 1%
40	Yvette Leung. "Exposing the Weaknesses: A Systematic Review of Azathioprine Efficacy in Ulcerative Colitis", Digestive Diseases and Sciences, 06/2008<br>Crossref	10 words — < 1%
41	www.oncotarget.com<br>Internet	10 words — < 1%

42 Arrigo F.G. Cicero, Alessandro Colletti. "Polyphenols Effect on Circulating Lipids and Lipoproteins: From Biochemistry to Clinical Evidence", Current Pharmaceutical Design, 2018 — 9 words — < 1%
Crossref

43 Muhammad Aziz, Swetha Gannarapu, Choudhry Humayun, Usman Siddiqui, Khurram Nasir, Ehimen C. Aneni. "Association of particles of lipoprotein subclasses with arterial stiffness in a high-risk working population: the Baptist Employee Healthy Heart Study (BEHHS)", The Egyptian Heart Journal, 2020 — 9 words — < 1%
Crossref

44 dmr.gov.mm — 9 words — < 1%
Internet

45 nrl.northumbria.ac.uk — 9 words — < 1%
Internet

46 rmdopen.bmj.com — 9 words — < 1%
Internet

47 www.bioseek.eu — 9 words — < 1%
Internet

48 Aya Hirata, Akemi Kakino, Tomonori Okamura, Yoko Usami et al. "The relationship between serum levels of LOX-1 ligand containing ApoAI as a novel marker of dysfunctional HDL and coronary artery calcification in middle-aged Japanese men", Atherosclerosis, 2020 — 8 words — < 1%
Crossref

49 Ki-Chul Sung, Sarah H. Wild, Christopher D. Byrne. "Controlling for apolipoprotein A-I concentrations changes the inverse direction of the relationship between high — 8 words — < 1%

HDL-C concentration and a measure of pre-clinical atherosclerosis", Atherosclerosis, 2013
Crossref

---

50 dliem.iums.ac.ir
Internet
8 words — < 1%

---

51 mijn.bsl.nl
Internet
8 words — < 1%

---

52 www.jlr.org
Internet
8 words — < 1%

---

53 Genevieve A. Woodard, Maria M. Brooks, Emma Barinas-Mitchell, Rachel H. Mackey, Karen A. Matthews, Kim Sutton-Tyrrell. "Lipids, menopause, and early atherosclerosis in Study of Women's Health Across the Nation Heart women", Menopause, 2011
Crossref
7 words — < 1%

---

54 Natalie D. Muth, Gail A. Laughlin, Denise von Mühlen, Sidney C. Smith, Elizabeth Barrett-Connor. "High-density lipoprotein subclasses are a potential intermediary between alcohol intake and reduced risk of cardiovascular disease: The Rancho Bernardo Study", British Journal of Nutrition, 2010
Crossref
7 words — < 1%

---

55 Sara Samadi, Mehrane Mehramiz, Theodoros Kelesidis, Majid Ghayour Mobarhan et al. "High‐density lipoprotein lipid peroxidation as a molecular signature of the risk for developing cardiovascular disease: Results from MASHAD cohort", Journal of Cellular Physiology, 2019
Crossref
7 words — < 1%

---

56 Wladimir M. Freitas, Luiz A. Quaglia, Simone N. Santos, Rafaela C. S. de Paula et al. "Low HDL
7 words — < 1%

cholesterol but not high LDL cholesterol is independently associated with subclinical coronary atherosclerosis in healthy octogenarians", Aging Clinical and Experimental Research, 2014
Crossref

57 Connie W. Tsao, Aaron W. Aday, Zaid I. Almarzooq, Cheryl A.M. Anderson et al. "Heart Disease and Stroke Statistics—2023 Update: A Report From the American Heart Association", Circulation, 2023
Crossref
6 words — < 1%

58 Farzaneh Asgari-Taee, Nahid Zerafati-Shoae, Mohsen Dehghani, Masoumeh Sadeghi, Hamid R. Baradaran, Shima Jazayeri. "Association of sugar sweetened beverages consumption with non-alcoholic fatty liver disease: a systematic review and meta-analysis", European Journal of Nutrition, 2018
Crossref
6 words — < 1%

59 Pagsberg, A. K., S. Tarp, D. Glintborg, A. D. Stenstrom, A. Fink-Jensen, C. U. Correll, and R. Christensen. "Antipsychotic treatment for children and adolescents with schizophrenia spectrum disorders: protocol for a network meta-analysis of randomised trials", BMJ Open, 2014.
Crossref
6 words — < 1%

60 Lena Tschiderer, Gerhard Klingenschmid, Rajini Nagrani, Johann Willeit et al. "Osteoprotegerin and Cardiovascular Events in High‐Risk Populations: Meta‐Analysis of 19 Prospective Studies Involving 27450 Participants", Journal of the American Heart Association, 2018
Crossref
5 words — < 1%

EXCLUDE QUOTES	ON	EXCLUDE SOURCES	OFF
EXCLUDE BIBLIOGRAPHY	ON	EXCLUDE MATCHES	OFF
